# A High Precision Feature Based on LBP and Gabor Theory for Face Recognition

**DOI:** 10.3390/s130404499

**Published:** 2013-04-03

**Authors:** Wei Xia, Shouyi Yin, Peng Ouyang

**Affiliations:** Tsinghua Center for Mobile Computing, Institute of Microelectronics, Tsinghua University, Beijing 100084, China; E-Mails: maxiaola@gmail.com (W.X.); oyangpeng12@163.com (P.O.)

**Keywords:** face recognition, new feature, robust, Gabor, LBP, PCA, LDA

## Abstract

How to describe an image accurately with the most useful information but at the same time the least useless information is a basic problem in the recognition field. In this paper, a novel and high precision feature called BG2D2LRP is proposed, accompanied with a corresponding face recognition system. The feature contains both static texture differences and dynamic contour trends. It is based on Gabor and LBP theory, operated by various kinds of transformations such as block, second derivative, direct orientation, layer and finally fusion in a particular way. Seven well-known face databases such as FRGC, AR, FERET and so on are used to evaluate the veracity and robustness of the proposed feature. A maximum improvement of 29.41% is achieved comparing with other methods. Besides, the ROC curve provides a satisfactory figure. Those experimental results strongly demonstrate the feasibility and superiority of the new feature and method.

## 1. Introduction

Face recognition has been investigated quite intensively in recent years owing to its wide range of applications in information security and access control, law enforcement, surveillance, and more generally, image understanding [1]. There are many face recognition methods, including Eigen faces and its modification [2], neural networks [3], and so on. Besides, the method based on model [4] is also popular with researchers. The ultramodern method involves compressed sensing and sparse basis data [5]. Most of these methods are feature-based, where the only difference is the type of features they choose. That is because the result of the recognition depends to a large extent on the feature selection. The most classical features are the Local Binary Pattern (LBP) [6] and Gabor feature. Numerous methods based on LBP or Gabor have been proposed to improve the recognition results [7–9]. Nevertheless, the bottlenecks people soon encountered in the face recognition, including gestures, expressions, and illuminations also became obvious and sharp [10]. Adini after an in-depth study conclued that illumination changes the appearance of a face drastically, and sometimes the differences may be even larger than those between different persons [11]. To solve this problem, lots of methods were proposed. Huafeng Qin et al. proposed a weighted region covariance matrix method based on the Gabor filter [12]. They construct a weighting matrix by computing the similarity of each pixel with a face sample first, and then develop a kernel Gabor-based weighted region covariance matrix. The result on the ORL database has demonstrated the advantages of this method. However, when tested on the Yale database, which includes many pose-variable pictures, its recognition rate is just 79.2%. The main reason may be though Gabor feature is robust towards illumination differences, it can't respond to expression or pose changes. Next Zhao and Zhang studied in depth the facial expression recognition using LBP and a Kernel discriminant isomap [13]. It obtains the best accuracy of 81.59% on the JAFFE database and 94.88% on the Cohn-Kanade database. This method makes sufficient use of the robustness of expression of LBP, but it can just recognize what expression the face shows while it may not recognize whom the face belongs to. Baochang Zhang et al. studied the LBP feature in depth and proposed an effective modification based on LBP in [14]. In their approach, they get a high-order local pattern descriptor (LDP), by encoding various distinctive spatial relationships contained in a given local region. This GLDP method performs well on the FERET and CAS-PEAL databases. However, most part of the features are useless and too spatial. Besides, the feature it used is only derivative, which just describes the general trend, is not accurate enough.

In the paper, we focus mainly on the issue of recognition accuracy, which without doubt also involves overcoming the challenges of lighting, pose, and expression variation. We propose a new robust feature called Block Gabor Directed Derivative Layer Local Radii-changed Binary Pattern (BG2D2LRP) after spending a lot of efforts in improving the weaknesses of existing features. The BG2D2LRP feature actually contains four aspects of information through series of transformations: the local region texture change trend, the local relations among three circle points. The feature not only preserves the essence of both the Gabor feature and LBP feature, but also results in excellent performance. Through those transformations, the BG2D2LRP feature overcomes the limitations of the Gabor feature that can only extract the spatial frequency and LBP that is not so good at discriminating between similar shapes. It is robust to the lighting, pose, and expression changes and even makeup embellishment. The operation of the BG2D2LRP feature is described step by step in Section 3.

This paper is organized as follows: Section 2 simply introduces the basic theory of the Gabor and LBP features. Then Section 3 specifically introduces our new feature. Its performance will be discussed and verified in detail. The related work to our approach will be mentioned in Section 4. Then the face databases we use and the experimental results are given in Section 5, in addition, the performance of our face recognition system is compared with other face recognition systems based on the same face databases. Section 6 gives some conclusions and future plans.

## 2. Backgrounds

The Gabor wavelet has been widely used to extract the texture features [15] since J.G. Daugmann declared that a simple cell could be approximated using 2D Gabor filters to the cells in the human virtual cortex and can be selectively related to orientation and to spatial frequency [16]. A detailed introduction of its performance is given in [17]. In this paper, we define the Gabor kernel function as follows:

(1) $$G=\frac{u^{2}+v^{2}}{{\text{δ}}^{2}}\cdot e^{-\frac{\left(u^{2}+v^{2})(x^{2}+y^{2}\right)}{2{\text{δ}}^{2}}}\cdot [e^{-i(\text{ux}+\text{vy})}-e^{-\frac{{\text{δ}}^{2}}{2}}]$$

where:

(2) $$k=\left(\begin{array}{l} u\\ v \end{array}\right);u=\frac{k_{\text{max}}}{f^{\text{Nu}}}\cdot \cos(\frac{\text{muπ}}{\text{δ}});v=\frac{k_{\text{max}}}{f^{\text{Nu}}}\cdot \sin(\frac{\text{muπ}}{\text{δ}})$$

Here u and v are the orientation and scale of the Gabor kernel. In most case, one would use Gabor wavelets at five different scales (Nu = 0,1,2,3,4), and eight different orientations (mu = 0,1,2,3,4,5,6,7). We define the parameters as follows:

(3) $$f=\sqrt{2},\text{δ}=2\text{π}$$

Given a gray-level image T(x,y), which has been pre-processed already, we take the convolution of it with a Gabor kernal as defined by function(4) for feature extraction and image representation:

(4) P(x,y)=T(x,y)∗G(x,y)

**Figure 1.**
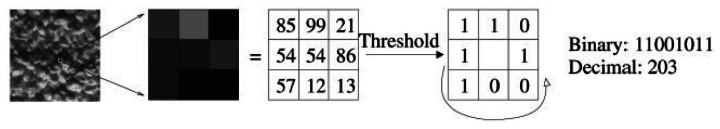
The processing of LBP operators.

Local Binary Patterns (LBP) was introduced as a texture descriptor by Ojala [18]. It labels a point as the center point, and compute the differences between it and the points around. On occasion that the difference is larger than 0, we assign the result to be 1, or else to be 0. We give an example in Figure 1.

**Figure 2.**
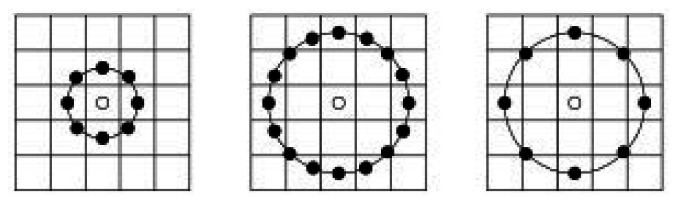
The circle LBP operators. (**Left**): R = 1.N = 8; (**Middle**): R = 2, N = 16; (**Right**): R = 2, N = 8.

Then the no-argument square region has been replaced by circle region and extended to consider different neighborhood sizes with two parameters R and N shown in Figure 2.

Here R means the region radius and N means the number of sampling points around the center point. Many methods based on LBP have been discussed, for example [19,20].

## 3. Layer Directed Derivative Local Radii-Changed Binary Pattern Feature (BG2D2LRP)

The Gabor feature and LBP feature have been successfully used in face recognition since they are robust to illumination and expression. However, they have some shortcomings: (a) they are too simple and vague for distinction since what they describe are only a rough and general outline. Many details as well as the unique information contained in an image have not been fully utilized; (b) although they overcome the influence of changing light to a certain extent, they are not so robust when faced with severe changes. Motivated by this, we thought the idea that we may not only use the static pixel local binary, but also the dynamic texture changes, and the latter one is even more important as it would be more unique for different persons. We compared Gabor values of each point with multiple nearby points to judge its shape and changing trend using the Double LBP model. The radii are set to be variable in order to identify the best radii for best performance (GLRP). We choose eight orientations unlike the traditional way of derivation [14], since directed orientation can enhance the distinguishability (directed). Then we take the dynamic changing trend of face along a set direction as its overarching characteristic, which demonstrates the unique contour information (derivative). The static differences are also used as a supplementary explanation. It should be noted that the images are divided into parts for better extraction performance as mentioned in [21] (blocking). After fusion by the layer method (layer), the feature (BG2D2LRP) which consists of both dynamic contour trends and static texture differences includes an almost substantially unique information for sample faces. More details are shown below.

**Figure 3.**
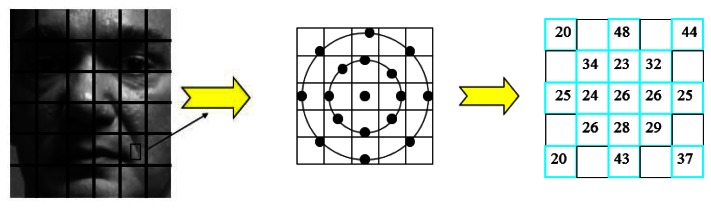
The Double Radii LBP model. N = 8, R1 = 2.5, R2 = 1.5. The left is the model and we mark the pixel value of the sampling points in the right.

First we chose a region that contains double circles both centered in a same point but with different radii. Each circle performs as mentioned in Section 2 and here we give N a constant value 8. The radii are variable, with different values and proportions. We use C1 and R1 to represent the outer circle and the corresponding radius, while C2 and R2 represent the inner circle and the corresponding radius. Figure 3 shows the Double Radii LBP model.

**Figure 4.**
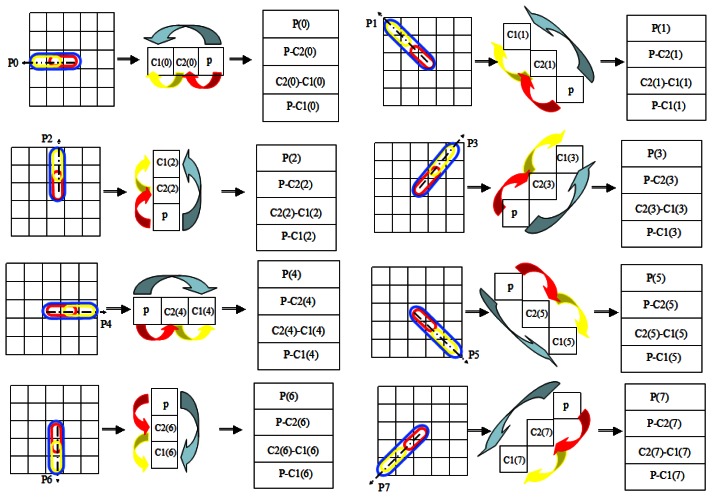
The principle of the layer directed derivative feature P0∼P7.

As we can see in Figure 3, when N = 8, there are just eight orientations clockwise from the center point: 0°, 45°, 90°, 135°, 180°, 225°, 270° and 315°, so we can compute the information of the local region in the way shown in Figure 4, which not only filters unnecessary information but also emphasizes the key information.

In Figure 4, there are eight iterations for a center point and each one takes three little blocks: (1) 5 × 5 box with three different ovals; (2) 1 × 3 rectangle lattices with three arrows; (3) 1 × 4 rectangle lattices. The oval region which covers three minimum square are extracted from the 5 × 5 double radii LBP model. The thick arrows indicate the operational relationship of squares in every oval region. Then we give the pixel value differences between the related squares in the 1 × 4 rectangle lattices.

Now each square get 4 × 8 features. Considering that the sign plays a more important role than the difference discussed in [22], we encode them with the rule:

(5) $${\text{code}}_{\left(\text{value}\right)}=\{\begin{array}{ll} 1 & \;(\text{value}>0)\\ 0 & \;(\text{else}) \end{array}$$

The code of P(i) is:

(6) $$P(i)={\text{Code}}_{\left(P-C2(i)\right)}⊙{\text{Code}}_{\left(C2(i)-C1(i)\right)}$$

Here *Code*_(*P*−*C*2(*i*))_ is the first derivative of the central square, and *Code*_(*C*2(*i*)−*C*1(*i*))_ is the surrounding changes of the center point. So *P*(*i*) can be regarded as the second directed derivative of the center square which describes the texture variation tendency along a certain orientation around it. If the code in it is “1”, it is monotonically increasing or decreasing from the center outward. On the contrary, if the code in it is “0”, it is first increased and then decreased or first decreased and then increased outwards from the center.

**Figure 5.**
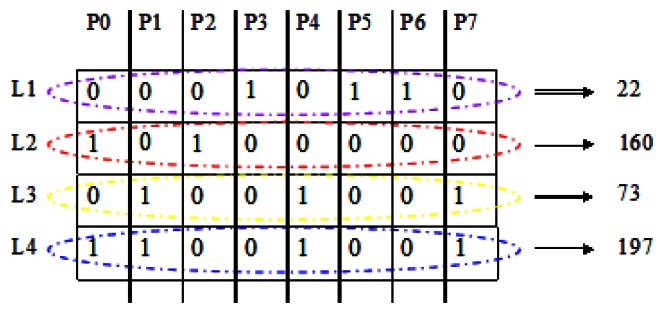
The finally new feature. Each column (P0∼P7) represents the sub-feature at different orientations of the center point. Each row (L1∼L4) represents the final BG2D2LRP feature of the center point.

Here we list the encoded features in turn just like the way shown in Figure 5. The detailed result in Figure 5 is an example based on the model in Figure 3. For computational convenience, we transfer the eight binary codes at each layer into a decimal number. These four decimal numbers contain almost all the required information for a square.

In Figure 5, L1 is the ensemble of *P*(*i*). It reflects the essential attributes of the texture distribution for a person, so it is very easy to distinguish person A from person B. However, it is vague sometimes, such as in the case of feature P1 and feature P2 in Figure 5, although their L1 value are both 0, in fact they represent contrary shapes.

That's why L2 and L3 are needed. The combination of L2 and L3 exactly overcomes this defect and assists L1 in pinpointing the change trend. L2 represents the difference between local region C1 and the center square, while L3 represents the difference the local region C1 and C2 next to the center square. They describe the change trend outward from the center point step by step, while L1 just describes the global monotone trend outwards from the center point. In other words, when “1” appears in L1, we will know clearly whether it is monotonically increasing or decreasing from the center outwards, according to the corresponding codes in L2 and L3.

L4 is the LBP code of the center point with a new radius. L4 and L2 make use of two kinds of LBP information at the same time. It is more comprehensive and distinguishable. We changed R1 and R2 in the experimental module and compared the results.

## 4. Our Approach

**Figure 6.**
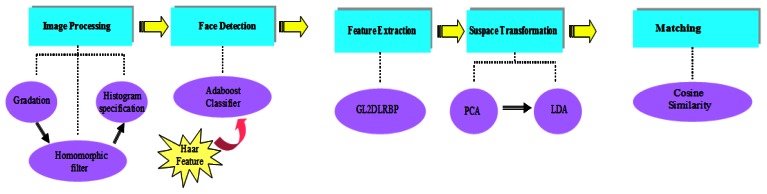
The recognition system of our approach.

Our approach can be illustrated in Figure 6. First we used a homomorphic filter and histogram specification to obtain an excellent image splicing effect. The Adaboost algorithm with Haar features [23] was applied to catch an accurate facial contour, which prepares for the BG2D2LRP feature extraction. After we have detected the face and resized the detected face to be 120 × 120, the BG3DLRP feature could be extracted from the face. We choose PCA [24] and LDA [25] for dimension reduction because they are useful to enhance the recognition performance of our feature. At last, given two vectors after PCA and LDA translation, we chose cosine similarity [26] to calculate the distance as it can effectively avoid the difference of the same individual in different degree and has better cooperation with BG3DLRP feature.

## 5. Experiments

### 5.1. Control Group Design

Considering our new feature is based on Gabor and LBP, we chose Gabor, LBP and LDP as comparison methods. To furthest improve recognition performance, we bring in PCA, LDA and cosine similarity and construct a mature face recognition system, so we take PCA, LDA into account for comparison. Besides, we compare our method with a similar method introduced in [12] to illustrate its advantages.

All code is written by C++ with OPENCV assistance. We reproduced those methods and do experiments in the same situations. The compared methods are all given the same pre-processing and cosine similarity operations. The differences lie in the feature extraction module and dimension reducing module. The detailed descriptions are as follows. Gabor method: Pre-processing + Haar-detection + Gabor + Cosine similarity; LBP method: Pre-processing + Haar-detection + LBP + Cosine similarity; GPCA method: Pre-processing + Haar-detection + Gabor + PCA + Cosine similarity; GLDA method: Pre-processing + Haar-detection + Gabor + LDA + Cosine similarity; GLDP method: Pre-processing + Haar-detection + Gabor + LDP + Cosine similarity; KGWRCM method: Pre-processing + Haar-detection + Gabor + PCA + Cosine similarity; Our Feature method: Pre-processing + Haar-detection + BG2D2LRP + Cosine similarity; Our Approach method: Pre-processing + Haar-detection + BG2D2LRP + PCA + LDA + Cosine similarity.

### 5.2. Database Sets

**Figure 7.**
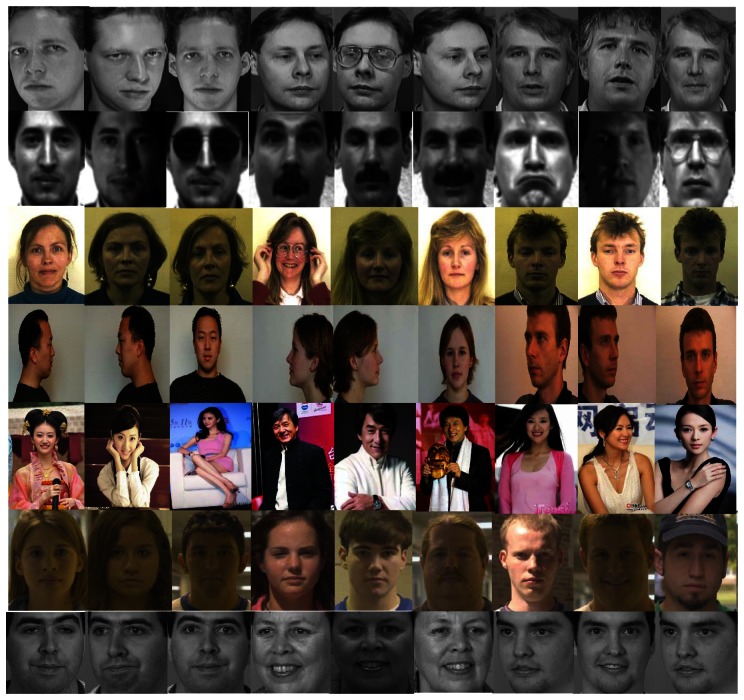
Databases. The six databases are, from top to bottom in turn: ORL, YALE, ABERDEEN, AR, FSTAR, FRGC, FERET (Reprinted with permission).

In the recognition module, we choose six well lnown databases and a self-made database to test our new feature. Each has its different emphasis, as shown in Figure 7. The ORL Database consists of 400 images of 40 different persons. The images mainly vary in pose and scale. The Yale Database contains 15 individuals with 11 images of each one. It emphasizes illumination and expression changes. There are 3,288 images corresponding to 116 people's faces in the AR Database which shows dramatic changes of lighting, expressions, poses and even accessories such as glasses and bangs. We also randomly choose three images of each person as training set and the rest as testing set. The ABERDEEN database includes 625 images of 65 persons. The images are strictly controlled in different lighting and expression conditions. The FRGC Database contains 12,776 images in the training set and 8,014 images in the testing set. This database is quite a challenge for its severe variation. The last database is FERET, which includes 1,400 cropped gray images of 200 persons. Angles and expressions are the focus. It should be noted that in our daily life, rather than the natural factors such as light or poses, makeup is the one that mostly distracting recognition problems. Noticing this fact, we downloaded images of film stars with different makeup from the internet and gather them together to form a database named FSTAR database. The FSTAR database contains 1,800 images of 120 persons. For each database, a random subset with three images per individual is used for training and the rest for testing. When the subset changed, we regard it as a new group of experiments. We performed thousands of groups of experiments with random subsets.

### 5.3. Results

**Figure 8.**
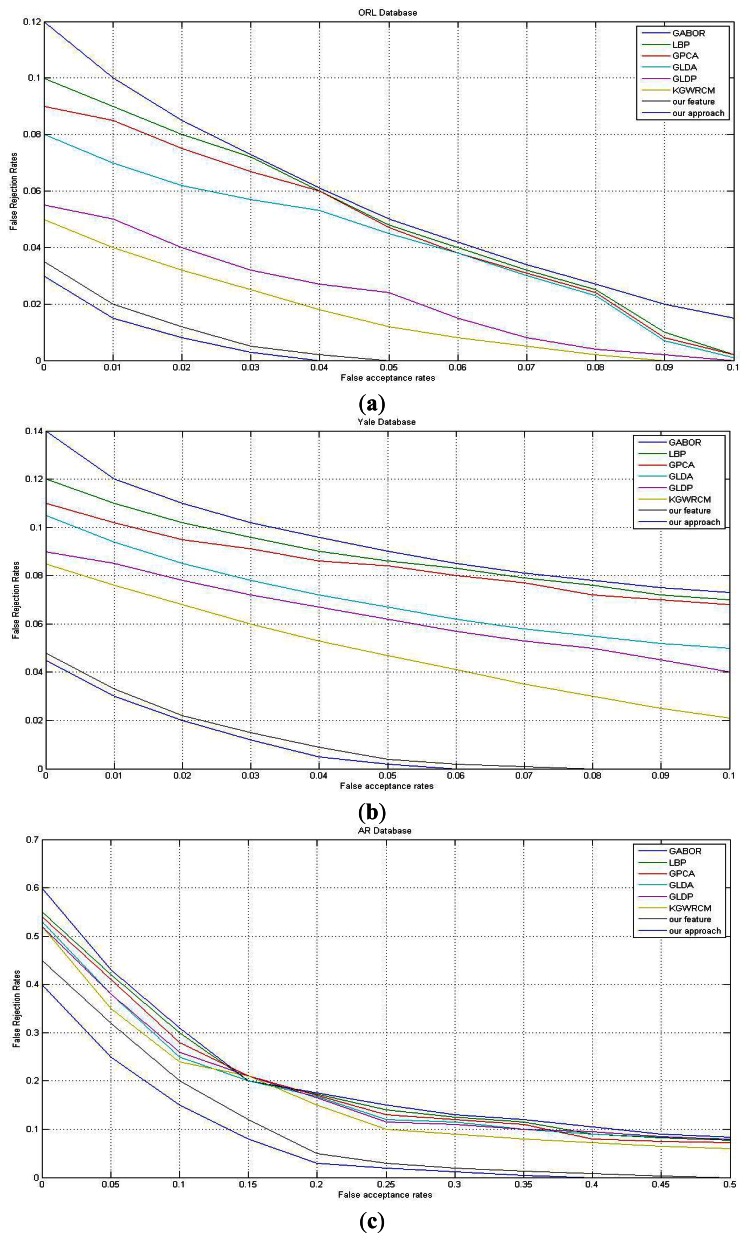

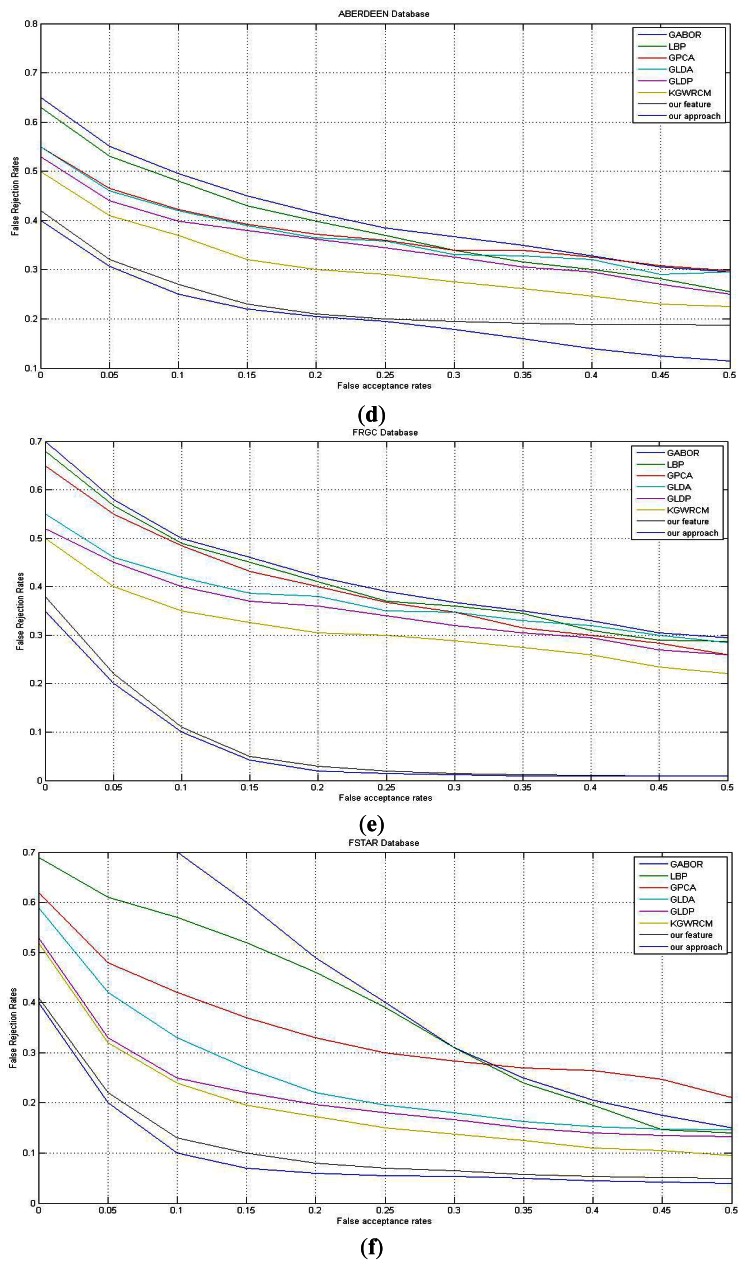

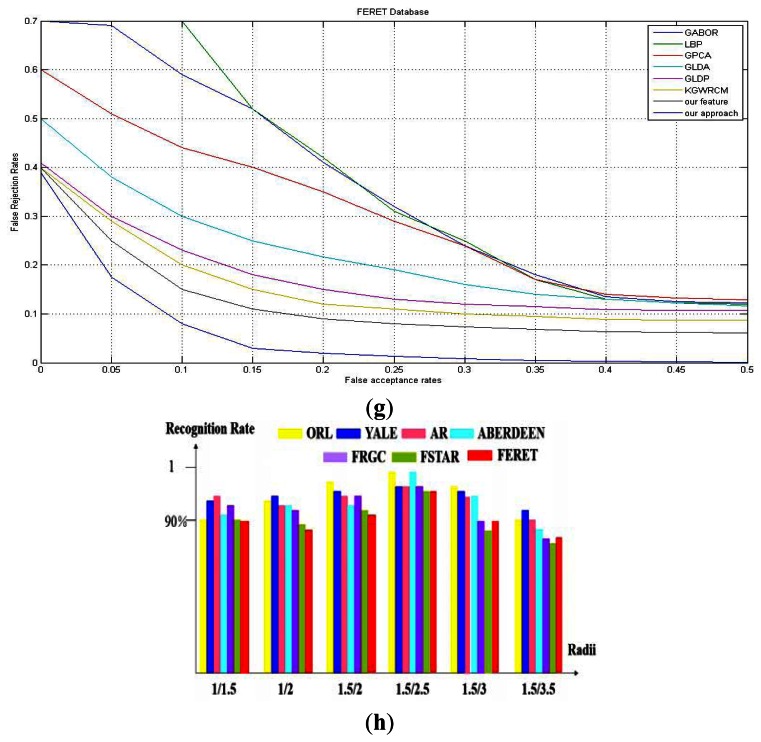
The ROC curve of different methods on databases. (**a**–**f**) in turn: ORL, YALE, AR, ABERDEEN, FRGC, FSTAR and FERET databases. (**g**) The recognition rate with different radii.

We measured three parameters as recognition performance indicators: Recognition Rate; Consuming Time; ROC Curve. The recognition rate is the most intuitive tool to indicate accuracy of the feature. We could get the cost of the feature and the identification system from the time consumption. In addition, the ROC curves illustrate the stability and robustness of the feature and system. The recognition rates are shown in Table 1 and Table 2. We gave the consumed time of each method in Table 3. The ROC curves are shown in Figure 8.

From Tables 1 and 2 we can see one of the major advantages of our new feature is that it can greatly improve the recognition rate. The recognition rate of our method always remains at more than 95%. We can see the recognition rates of most methods on the YALE database are even lower than of FRGC or FERET although YALE seems relatively simple.

The reason for this lies in the fact that many images in it involve partial occlusions on eyes or noses and thus provide limited information and strong interference, which is shown in Figure 7. However, the recognition rate of our feature and our approach can also reach 96.98% and 97.27% in these cases. Besides, the recognition rate of GLDP method also is 83.42%, which is more than PCA, KGWRCM. This demonstrates that the dynamic contour trend is more effective than static texture differences. There is almost a 12% improvement with our feature based on the GLDP method. This mainly results from two aspects: (a) our directed dynamic changing trend feature is better than the aimless LDP; (b) our static texture difference feature provides extra useful information to some extent.

Expression variation is really a challenge for face recognition. However, we can see the recognition rate of our approach can still reach 95.11% and 97.28% on FERET with our feature. Actually, expression changes can be regarded as a partial translation in the original two-dimensional (x,y) image. This means, the translation would only change the point value but not the shape. LBP is popular in expression recognition as these changes can be eliminated after subtraction. Considering our feature, on the one hand it contains a double LBP texture difference, which provides more information, and on the other hand the dynamic changing trend of it keeps stable no matter how expression varies, so it is markedly robust to expression changes.

Our feature performs well even with single eye and side face, as it extracts the most intrinsic characteristicd of the image, which solves the recognition pose variation problem. The recognition rate of our feature on the AR database which is famous for its severe pose changes is 96.32%, which also illustrates this thesis. The ABERDEEN database is made under strict light changing conditions. Our feature is insensitive to illumination as the difference operation eliminated the illumination changes. As for the FRGC and FSTAR databases, which have complicated changes, our feature is even superior. It shows significant improvement over the existing relative methods. Besides, from the data, we can see that the difference of our feature and our approach is always less than 1%. This means although our approach would improve the recognition rate, the perfect result should be due to our accurate feature.

We need ROC curve to evaluate the recognition performance while recognition rate just illustrates the result accuracy. As we all know, the smaller the graphics area between the curve and the coordinate axis, the better the method. In Figure 8, the curves of our method and feature are always in the bottom far below the curves of other methods. They are near and even overlapped in some cases. This also validates the conclusion that the perfect result should be due to our accurate feature. It remains gradual even when tested on the FRGC database, in which curves of other methods vary sharply. This should be attributed to the L2 and L3 part of our new feature. Although the light changes, the difference would remain unchanged as the variation errors are eliminated by subtracting. The expression robustness mainly is due to the L1 part of the new feature. It catches the texture changing trend of a person, although the expression may change the pixel gray value, the value changing trend within a local region may remain stable. All results of our new feature and approach show impressively superiority to the results of LBP, Gabor features and other similar methods.

In general, ORL, YALE, AR are relatively simple, the recognition rates on it are high and the ROC curves are better than on FERC, FERET, FSTAR, ABERDEEN databases. It should be noted that the more difficult the databases, the greater the difference between our feature and other methods. This means our feature can bear changes more widely than other methods. The joint effort of static texture difference and dynamic contour consists of our high accuracy feature which extracts maximum intro-class similarity and inter-class otherness information.

Besides, when testing with our methods, we sought to find how the local region size would impact the recognition rate, thus we conducted a new group of experiments by changing the radii of C1 and C2 each time and recording the result data. The result of this experiment is shown in Figure 8(h). We can see that the radius should be neither too big nor too small. It's the best choice when the two circles are just the adjacent layers around the center point. If the radii are both too small, the two circles are too near and the information is not representative, especially after subtraction. In contrast, if the local region is too sparse, the information of those pixels would have no rules or connections.

Table 3 gives the time consumed by each method per 300 images. It refers to the average time that training samples need to form a characteristic template. In the table we find that Gabor feature and LBP feature are fairly fast, however, PCA and LDA takes more time as they involved in complicated matrix operations. Our feature takes only 4.1 s, even though it contains many operations. This is due to the manipulations of our feature are simple subtraction and logical calculus, which consume less time than matrix operations.

## 6. Conclusions

In this paper, we introduced a new feature for face recognition. It absorbs the essence of the Gabor and LBP features, and contains static texture differences and dynamic contour trends, so it reflects the substantive characteristics of the facial texture features. It remains robust to light, expression, and pose changes and makeup. We tested the feature on seven well known databases. The results show that the recognition rate is always more than 95%, giving us confidence that our feature can tremendously improve the quality of face recognition. Furthermore, it takes only 4.1 s to accomplish the whole feature extraction program. However, there are still some deficiencies could be improved in our approach. From the ROC curve, we find the unrecognized images are mainly on account of the undetection. Then in the future, some improvements to the detection aspect may be researched. Moreover, we will initiate modifications on the BG2D2LRP feature, such as finding the optimal radii for each database automatically, or changing the pixels on the circles, in the purpose of improving the recognition rate. A sparse representation classification model is researched to place of PCA+LDA+Cos Similarly, we believe it would improve a lot of our features. In addition, we will turn to 3D face recognition by adding depth information for better recognition performance.

## Figures and Tables

**Table 1.** The Recognition Rate (%) based on ORL, YALE, and AR Database.

**Methods**	**ORL**	**YALE**	**AR**
Gabor	81.34	66.78	78.64
GPCA	89.78	67.94	88.99
GLDA	97.50	73.47	75.95
LBP	89.61	78.85	84.31
GLDP	93.42	83.42	90.11
KGWRCM	99.32	80.20	95.95
**Our Feature**	**99.47**	**96.98**	**96.34**
**Our Approach**	**99.75**	**97.27**	**96.82**

**Table 2.** The Recognition Rate (%) based on ABERDEEN, FRGC, FSTAR and FERET Databases.

**Methods**	**ABERDEEN**	**FRGC**	**FSTAR**	**FERET**
Gabor	70.27	73.91	67.85	66.93
GPCA	73.86	78.95	65.81	72.35
GLDA	78.65	82.89	70.32	74.19
LBP	78.59	79.15	68.55	78.44
GLDP	83.29	89.72	75.67	90.32
KGWRCM	85.78	91.45	83.15	92.07
**Our Feature**	**99.03**	**95.09**	**94.94**	**95.11**
**Our Approach**	**99.68**	**95.27**	**95.24**	**97.28**

**Table 3.** The time consumed of each method per 300 images.

**Methods**	**Gabor**	**LBP**	**GPCA**	**GLDA**	**GLDP**	**KGWRCM**	**OURS'**
Feature Time(s)	1.8	1.5	7.2	6.9	3.7	4.2	**4.1**
